# Genetic basis of common human disease: insight into the role of nonsynonymous SNPs from genome-wide association studies

**DOI:** 10.1186/gb-2011-12-s1-p10

**Published:** 2011-09-19

**Authors:** Lipika R Pal, John Moult

**Affiliations:** 1 Institute for Bioscience and Biotechnology Research, University of Maryland at College Park, Rockville, MD 20850, USA; 2 Department of Cell Biology and Molecular Genetics, University of Maryland at College Park, College Park, MD 20742, USA

## Background

Recent genome-wide association studies have led to the reliable identification of single nucleotide polymorphisms (SNPs) at a number of loci associated with an increased risk of developing specific common human diseases. Each such locus implicates multiple possible candidate SNPs as being involved in the disease mechanism, and determining which SNPs actually contribute, and by what mechanism, is a major challenge. A variety of mechanisms may link the presence of a SNP to altered *in vivo* gene product function and hence contribute to disease risk. We have analyzed the role of one of these mechanisms, nonsynonymous SNPs (nsSNPs) in proteins, for associations found in the Wellcome Trust Case-Control Consortium (WTCCC) study of seven common diseases [1] and the follow-up work.

## Methods

Using HapMap data and linkage disequilibrium information, we identified all possible candidate SNPs associated with increased disease risk. We then applied two computational methods [2,3], based on analysis of protein structure and sequence, to determine which of these SNPs has a significant impact on *in vivo* protein function (SNPs3D) [4].

## Results

Several of these disease-associated loci were found to be linked to one or more high-impact nsSNPs. In some cases, these SNPs are in well-known proteins (such as human leukocyte antigens). In other cases, they are in less well-established disease-associated genes (for example, MST1 for Crohn’s disease), and in yet others, they are in proteins that have been poorly investigated (for example, gasdermin B, also for Crohn’s disease). Approximately 55% of these disease-associated loci have at least one nsSNP, and about 33% of them have at least one high-impact nsSNP in those regions.

## Conclusions

Together, these data suggest a significant role for nsSNPs in common human disease susceptibility.
